# Evaluating the Antibacterial Activity and Mode of Action of Thymol-Loaded Chitosan Nanoparticles Against Plant Bacterial Pathogen *Xanthomonas campestris* pv. *campestris*

**DOI:** 10.3389/fmicb.2021.792737

**Published:** 2022-01-14

**Authors:** Sarangapani Sreelatha, Nadimuthu Kumar, Tan Si Yin, Sarojam Rajani

**Affiliations:** ^1^Temasek Life Sciences Laboratory, National University of Singapore, Singapore, Singapore; ^2^Department of Biological Sciences, National University of Singapore, Singapore, Singapore

**Keywords:** nanoparticles, antibacterial activity, volatiles organic compounds, mode of action, biofilm inhibition

## Abstract

The bacterium *Xanthomonas campestris* pv. *campestris* (*Xcc*) causes black rot disease in cruciferous crops, resulting in severe yield loss worldwide. The excessive use of chemical pesticides in agriculture to control diseases has raised significant concern about the impact on the environment and human health. Nanoparticles have recently gained significant attention in agriculture owing to their promising application in plant disease control, increasing soil fertility and nutrient availability. In the current study, we synthesized thymol-loaded chitosan nanoparticles (TCNPs) and assessed their antibacterial activity against *Xcc.* The synthesis of TCNPs was confirmed by using ultraviolet–visible spectroscopy. Fourier-transform infrared spectroscopy, transmission electron microscopy, and scanning electron microscopy analysis revealed the functional groups, size, and shape of TCNPs, with sizes ranging from 54 to 250 nm, respectively. The antibacterial activity of TCNPs against *Xcc* was investigated *in vitro* by liquid broth, cell viability, and live dead staining assay, and all of them demonstrated the antibacterial activity of TCNPs. Furthermore, TCNPs were found to directly inhibit the growth of *Xcc* by suppressing the growth of biofilm formation and the production of exopolysaccharides and xanthomonadin. The ultrastructure studies revealed membrane damage in TCNP-treated *Xcc* cells, causing a release of intracellular contents. Headspace/gas chromatography (GC)–mass spectrometry (MS) analysis showed changes in the volatile profile of *Xcc* cells treated with TCNPs. Increased amounts of carbonyl components (mainly ketones) and production of new volatile metabolites were observed in *Xcc* cells incubated with TCNPs. Overall, this study reveals TCNPs as a promising antibacterial candidate against *Xcc.*

## Introduction

To meet the global food security challenge, a substantial increase in crop production is required. Plant disease and pests cause significant yield loss, posing a great threat to agricultural productivity (Balaure et al., 2017). Pest and disease management in agriculture currently relies on the use of pesticides, but there are several issues with regard to the heavy usage of synthetic pesticides, such as the emergence of resistant pathogens and their harmful effects on the environment and human and animal health. In recent years, consumption of green leafy vegetables has increased immensely due to their high nutritional and antioxidant value. The genus *Brassica* contains many important vegetable crops, but they are highly susceptible to various pathogens such as bacteria, fungi, and nematodes, causing huge production losses. Black rot disease caused by the Gram-negative bacterium *Xanthomonas campestris* pv. *campestris (Xcc*) is a significant disease of all *Brassica* crops worldwide (Vicente and Holub, 2013; Nuñez et al., 2018). Synthetic pesticides such as quinolones, benzimidazole derivatives, dithiocarbamate, copper substances, and antibiotics are commonly used to control the black rot disease in vegetable crops, but their efficacy in controlling the disease spread is low (Patyka et al., 2016). Actigard, a chemical that induces a defense response in plants, is also used to control *Xcc* infection, but its effectiveness among different crops varies (Yuliar et al., 2015). Given the mutagenic, carcinogenic, and allergenic effects of synthetic pesticides on biological objects, the development of new biocontrol agents with better specificity of action, less toxicity, and without any adverse effects on the environment is required (Van Bruggen and Finckh, 2016).

As a result, substantial research efforts are being made towards the identification of new antimicrobial agents, which can be formulated into eco-friendly pesticides (Bhardwaj et al., 2014). Nanotechnology has gained momentum in medicine, pharmacology, engineering, food, and agrochemical industry with a wide range of applications. One of the significant interests of using nanotechnology in agriculture is to reduce the amount of agrochemicals used and to increase the yield through better pest and nutrient management. Engineered nanoparticles with desired characteristics can be used as carriers for the delivery of new generation of pesticides for effective plant disease management (Ivo et al., 2017). In the recent past, the agriculture sector has witnessed an expeditious development of nano-pesticides as an alternative to chemical pesticides where the efficacy of biocontrol agents is enhanced by converting them into nanoparticles or conjugating them with nanoparticles with reduced toxicity and controllable composition. Furthermore, these formulations can also be used to increase the shelf life of agricultural produce (Ishkeh et al., 2021; Kutawa et al., 2021; Zobir et al., 2021).

Chitosan is the second most abundant polymer, and its unique characteristics such as biodegradability, amenable biological properties, and non-toxicity against humans make it suitable for various potential applications in diverse fields. It can be used as a carrier to attach or encapsulate active reagents to develop agricultural formulations. Chitosan is a natural antimicrobial agent and found effective against a variety of bacteria and fungus (Meng et al., 2020; Kutawa et al., 2021). However, its efficacy is highly dependent on the type of microorganisms. The mechanism of the antimicrobial activity of chitosan are largely dependent on its physiochemical properties (Ke et al., 2021). Chitosan in the form of submicron dispersion showed antifungal potential against *Colletotrichum gloeosporioides*, which causes anthracnose of fresh fruits. Submicron chitosan dispersions were found to decrease the production of the cell wall degrading enzymes of *C. gloeosporioides* (Zahid et al., 2014, 2015). Additionally, chitosan-based coatings and packaging increased the shelf life of fruits (Ali et al., 2013; Ishkeh et al., 2021).

Previous studies have shown that chitosan is effective *in vitro* against *Xanthomonas axonopodis* pv. *poinsettiicola* isolated from *Euphorbia pulcherrima*. It was found that membrane damage and biofilm disruption played an important role in the antibacterial mechanism of chitosan (Li et al., 2008; Chang et al., 2012). The antimicrobial activity of chitosan can be enhanced by complexing it with suitable materials. Encapsulation of plant essential oils in chitosan-based coatings is gaining interest in agriculture research due to the antimicrobial properties associated with these volatile organic compounds (VOCs). Recently, several different plant essential oils have been incorporated into chitosan, and their antimicrobial activity was shown against a wide range of microbes (Rabea et al., 2003). Thymol, a natural monoterpenoid VOC found in the essential oil of *Thymus vulgaris* plant, is a potent antimicrobial agent either alone or in combination with other compounds (Chen et al., 2015). The antibacterial activity of thyme oil and thymol against a few *Xanthomonas* species has been reported (Singh et al., 2017). To increase the potency and stability of thymol, its encapsulation with chitosan was investigated. It was shown that thymol-based chitosan nanogels demonstrated significant antioxidant activity (Ghaderi Ghahfarokhi et al., 2016). The antibacterial effect of thymol-loaded chitosan nanoparticles (TCNPs) has been reported against a broad spectrum of Gram-positive and Gram-negative pathogens (Hu et al., 2009). Furthermore, the inhibitory activity of thyme essential oil-loaded chitosan nanoparticles and nanocapsules has been reported against many foodborne bacterial pathogens (Pecarski et al., 2014). These reports interested us in developing active TCNPs and exploring their activity against *Xcc*, which is a devastating disease in cruciferous vegetables. Although there are several reports on the antibacterial effect of chitosan and thymol either alone or together, very few studies have evaluated in-depth active mechanisms of their action, which would greatly facilitate the development of future eco-friendly nanopesticides.

In this study, we synthesized TCNPs and characterized them by ultraviolet–visible (UV-Vis) spectroscopy, high-resolution transmission electron microscopy, scanning electron microscopy (SEM), and Fourier-transform infrared spectroscopy (FTIR). The particle size and charge were determined by dynamic light scattering (DLS) and zeta potential analysis. Besides this, its bactericidal activity against *Xcc* was analyzed by multiple biochemical assays. Furthermore, the synthesized nanoparticles were examined for their mechanism of antibacterial action through spectroscopy, microscopy, membrane potential studies, and volatile analysis. Altogether, the present work provides a comprehensive analysis of the antibacterial action of the eco-friendly chitosan–thymol conjugate nanoparticles towards controlling the bacterial disease of cruciferous vegetables.

## Materials and Methods

### Chemicals and Bacterial Strain

Nutrient agar, broth media, chitosan low molecular weight (50–190 KDa) (from shrimp cells, 75–85% degree of deacetylation), thymol, and sodium tripolyphosphate were purchased from Sigma-Aldrich Co. (MO, United States). *Xcc* used in the current study was obtained from and cultured as per the protocol prescribed by the American Type Culture Collection (33913). Initially, bacteria were streaked from −80°C glycerol stock onto a nutrient agar plate, and a single colony was inoculated into yeast extract, glucose, and calcium carbonate (YGC) media and incubated at 28°C for 24 h. From there, 10^8^ CFU/ml bacterial cell suspensions were taken for all subsequent experiments.

### Synthesis, Characterization, and Loading Efficiency of Thymol-Loaded Chitosan Nanoparticles

TCNPs were prepared according to the method described previously (Hu et al., 2009) with some modifications. Briefly, chitosan (1%) was dissolved in an aqueous 1% acetic acid solution, and it was stirred at 300 rpm overnight at 25^°^C. After the adjustment of pH to 4.6 with continuous stirring for 30 min, the resulting clear solution was mixed with (1%) thymol and stirred for 3 h at 25^°^C with a few drops of tripolyphosphate (0.1%) solution added. After stirring, the resulting solution was centrifuged at 10,000 rpm for 30 min at 4^°^C, washed several times with distilled water, and then sonicated for 3 h at room temperature. The TCNP sample suspension obtained was studied for shape, size distribution, surface charge, and functional groups by SEM, DLS, zeta potential analysis, and FTIR as per the methods described in Das et al. (2012). The loading efficiency of thymol was analyzed as described in Venkatesan et al. (2016). The sample was centrifuged at 12,000 rpm to separate the pellet from the supernatant. The amount of thymol in the supernatant was determined by using UV-Vis spectrophotometer at 328 nm. The amount of thymol-loaded was calculated by subtracting the amount of thymol in the supernatant from the total amount of thymol used in the formulation.

### *In vitro* Antibacterial Activity of Thymol-Loaded Chitosan Nanoparticles

The antibacterial activity of TCNPs was determined against the *Xcc* in liquid broth by measuring the optical density (OD) at 600 nm (Ahmed et al., 2021). Briefly, mid-logarithmic phase, bacteria were cultured in YGC broth and then diluted to 10^8^CFU/ml. Two hundred microliters of the bacterial culture with different concentrations of TCNPs (100–600 μg/ml) were added to each well of the sterile 96-well plate. After incubation for 24 h at 28°C, minimum inhibitory concentrations were determined as the lowest concentration of compounds that prevented visible turbidity by visual inspection. The tests were performed in triplicates. The OD value at 600 nm was recorded using a Tecan (Infinite series, M200 pro) plate reader. It is reported as their minimum inhibitory concentration, which is the lowest concentration of the antibacterial agent required to inhibit the growth of a microorganism after overnight incubation.

### Cell Viability Assay

To measure the cytotoxicity of TCNPs against *Xcc* bacterial cells by the method described in Sahoo et al. (2016), 3-(4, 5-dimethylthiazol-2-yl)-2, 5-diphenyl tetrazolium bromide (MTT) assay was performed. Briefly, bacterial culture of 10^6^CFU/ml was added to the microtiter plate with TCNPs at different concentrations and incubated at 37°C for 24 h. The relative number of viable cells was then determined by adding 1 mg/mL of MTT and incubated further for 4 h at 37°C. The formazan crystals formed were then dissolved in dimethylsulfoxide, and the absorbance intensity was measured at 570 nm using a Tecan plate reader, which directly represents the relative cell numbers. All the experiments were performed in triplicates, and the cell viability was expressed as percent relative to the untreated control cells.

### Intracellular Reactive Oxygen Species Assay

Reactive oxygen species (ROS) generated in the bacterial cells after treatment with TCNPs was analyzed using the fluorescent probe 2′,7′-di-chlorofluorescein diacetate (DCFH-DA) as described in Bondarenko et al. (2012). Briefly, cells were grown overnight at 28°C at 150 rpm for 24 h. The bacterial cell suspension was then adjusted to 10^8^ CFU/ml, and 100 μl was transferred into each well of the 96-microtiter plate and treated with different concentrations of TCNPs for 24 h. After 24-h treatment, the cells were harvested by centrifugation at 3,500 rpm for 5 min at 4°C, washed with PBS, and incubated with 10 μM of DCFH-DA for 30 min at 37°C in the dark. A negative control comprising cells without treatment was also labeled with 10-μM DCFH-DA. The fluorescence intensity was measured using a Tecan microplate reader with excitation at 485 nm and emission at 530 nm. The results were expressed as a percentage of ROS with respect to control.

### Morphological Changes of Bacterial Cells by Scanning Electron Microscopy

Morphological changes of the bacterial cells (with and without TCNPs for 12 h) were fixed with 2.5% glutaraldehyde solution overnight at 4°C. The cells were then dehydrated with 50, 70, 80, 90, and 100% ethanol for 20 min each. The surface morphology of the prepared bacterial cells was analyzed by mounting the samples on SEM Stub mount coated with carbon and visualized by high-resolution SEM (JSM 6310, Jeol Ltd., Akishima, Tokyo, Japan) operated at 10-Kv voltage (Mahdi et al., 2020).

### Live/Dead Bacterial Staining Assay

The live/dead staining assay was used to determine the membrane damage of bacteria (Cui et al., 2014). Briefly, bacterial culture (10^8^ CFU/ml) was incubated with 500 μg/ml of TCNPs for 12 h, and then, it was centrifuged at 8,000 × *g* for 5 min, and the supernatant was discarded. The live bacterial cells without TCNPs were used as the control. The staining reagent mixture, a red fluorescent propidium iodide (PI) stain and a green fluorescent (SYTO 9) stain, was added to the reaction mixture and incubated in the dark at a room temperature for 15 min. The fluorescence emission of bacteria was assessed by means of confocal laser scanning microscopy (Olympus inverted confocal).

### Biofilm Inhibition Assay

The inhibition of *Xcc* biofilms by TCNPs was measured in a 96-well microtiter plate method as described previously (Ogunyemi et al., 2020). Briefly, 100 μl of bacterial cells (approximately 10^8^ CFU/ml) was inoculated in each well with TCNPs at different concentrations and incubated at 28^°^C for 24 h without agitation for the adhesion. Bacterial culture without TCNPs was used as the control. Culture media were removed and washed gently with sterile distilled water. Crystal violet solution (0.1%, w/v) was added to stain the biofilm and then kept for incubation for 30 min at room temperature. The unattached crystal violet solution was discarded, and then, the crystal violet stain was dissolved using 33% acetic acid, and the absorbance was measured at 570 nm using a Tecan microplate reader.

### Confocal Imaging

Briefly, *Xcc* was allowed to grow on the coverslips placed in 24-well polystyrene plates supplemented with and without TCNPs (500 μg/ml), incubated for 24 h at 28°C, and stained with acridine orange solution (v/v) for 20 min at room temperature. The biofilms stained with acridine orange were imaged using confocal microscopy (Olympus) at 40 × magnification. The 488-nm Ar laser and a 500–640-nm bandpass emission filter were used to excite and detect the stained cells. Confocal laser scanning microscopy images were obtained from the 24-h-old control and treated biofilms (Liu et al., 2015).

### Quantitation of Exopolysaccharide Production and Xanthomonadin

For the measurement of exopolysaccharide (EPS) production, *Xcc* cells were grown with and without TCNPs (500 μg/ml) in YGC broth at 28°C for 72 h. Cells were removed by centrifugation at 15,000 × *g* for 12 min, and the supernatant was collected for further analysis. To the collected supernatant, 1% potassium chloride and twice the volume of 100% ethanol were added and further incubated at −20°C overnight. The precipitated EPS was collected by centrifugation and dried overnight at 55°C, and the total dry weight was measured. The production of EPS was quantified relative to the cell density (Wang et al., 2015). For the quantitation of xanthomonadin pigment, *Xcc* cells with and without TCNPs (500 μg/ml) were collected by centrifuging 4 ml of broth suspension to which 100% methanol was added. The cell mixture was further incubated in darkness for 10 min in a rotating shaker followed by centrifugation at 12,000 × *g* for 8 min to collect the supernatant. Xanthomonadin pigment was quantified by measuring the absorbance at OD 445, and the result was expressed relative to the cell density measured before the assay at OD 595 (Wang et al., 2015).

### Membrane-Active Mechanism of Action

#### Inner Membrane and Outer Membrane Permeabilization

Outer membrane permeabilization was examined using the 1-N-phenylnaphthylamine (NPN, Sigma) uptake assay (Gerits et al., 2016). Briefly, 150 μl of bacterial suspension (10^8^ CFU/ml) in 5-mM 4-(2-hydroxyethyl)-1-piperazineethanesulfonic acid was treated with TCNPs at different concentrations and transferred to a 96-well microtiter plate. To this, 40 μm of NPN in 5-mM 4-(2-hydroxyethyl)-1-piperazineethanesulfonic acid was added, and the fluorescence was measured immediately using a Tecan microplate reader. The fluorescence was monitored at an excitation wavelength of 622 nm and an emission wavelength of 670 nm for both treated and untreated samples. The increase in the fluorescence was measured for 15 min for the membrane permeabilization at 37°C. All experiments were performed in triplicates, and the results are expressed in relative fluorescence units. Likewise, inner membrane permeabilization was examined using PI uptake assay (Gerits et al., 2016). PI dye was added to the bacterial suspension and incubated for 20 min. The fluorescence was monitored for 30 min at an excitation wavelength of 535 nm and an emission wavelength of 617 nm for both treated and untreated samples using a Tecan microplate reader. The uptake of the PI dye denotes the permeability of the cell membrane as detected by an increase in fluorescence. All experiments were performed in triplicates, and the results are expressed in relative fluorescence units.

### Volatile Changes in Microbial Interactions

*Xcc* cells were cultured in glass vials in a YGC medium with and without TCNPs (500 μg/ml) and incubated at 28°C for 24 h. The vials containing the YGC medium, control cells, and the treated cells were analyzed by the headspace solid-phase microextraction–GC-MS method (Monedeiro et al., 2021). A divinylbenzene-carboxen polydimethylsiloxane (50/30 μm) fiber (Supelco, Bellefonte, PA, United States) was exposed to the headspace of the samples for 40 min at 37^°^C. The isolated VOCs were analyzed by GC-MS on GC coupled to a 7200B MS detector (Agilent Technologies, Palo Alto, CA, United States). VOCs were desorbed by insertion of the solid-phase microextraction fiber into the GC injection port, in splitless mode, for 2 min at 250^°^C. The compounds were separated on an HP-5MS capillary column (30 m length, 0.25 mm id, 0.25 mm), using helium as carrier gas at 1 ml/min. The oven temperature was 40^°^C for 2 min, then increased to 240^°^C at 6 C/min and held for 5 min. MS detector was operated in electron impact mode (*EV* = 70 eV), in scan mode from 30 to 550 m/z, with an ion source temperature of 250^°^C. VOCs were identified by comparing their MS spectra with the National Institute of Standards and Technology library spectral databases and using authentic standards when available. VOC compounds identified in vials not inoculated were excluded from the data analysis. The relative quantities of the volatile compounds are expressed as percent peak areas relative to the total peak area of the identified compounds from the average of the three replicates.

#### Data Analysis

All the parameters studied were subjected to statistical treatment using SPSS statistical package (version 23.0). Data are represented as mean ± standard deviation of three independent experiments, each performed in triplicates. One-way analysis of variance was adopted to all the parameters under study to test the level of statistical significance. The difference was considered significant if *p < 0.05.

## Results and Discussion

### Synthesis and Characterization of Thymol-Loaded Chitosan Nanoparticles

**FIGURE 1:**
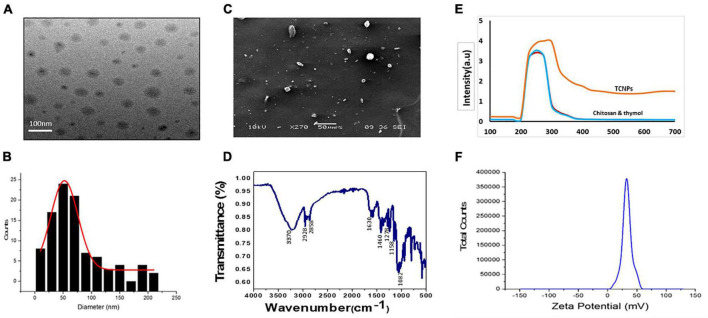
Characterization of thymol loaded chitosan nanoparticles. (TCNPs). **(A)** TEM analysis **(B)** Histogram for particle size distribution by DLS **(C)** SEM analysis **(D)** FTIR spectrum for thymol loaded chitosan nanoparticles. **(E)** UV—visible absorption spectrum of the nanoparticles **(F)** Zetapotential for the nanoparticles.

TCNPs were synthesized, and the formation of the nanoparticles was visible as an opaque solution, which was also confirmed by UV-Vis spectrophotometer, which showed an absorption maximum at 300 nm. The particle size distribution and the zeta potential of TCNPs were measured by DLS and Malvern zetasizer, as shown in Figures 1B,E,F. The average size of TCNPs suspension was in the range of 54–250-nm size with 70% loading efficiency of thymol in chitosan nanoparticles. Thymol release is directly dependent on the loading efficiency, which enhances a sustained release of thymol. The zeta potential value of TCNPs was found to be 45.54 mV, which denotes that TCNPs have good stability in water due to their electrostatic repulsion mechanism. This is in accordance with the study reported in Arulmozhi et al. (2013), who showed that a high zeta potential greater than 30 mV makes the nanoparticles repel each other, which ensures physical colloidal stability of the suspensions. No phenomenon of aggregation and flocculation was detected in TCNPs, denoting its stability further.

FTIR analysis of TCNPs was performed to identify the interaction between the molecules. Several dominant peaks were observed in the FTIR spectra. Peaks in the range of 2,858.54 and 2,928.83 cm^–1^ are associated with the C-H bond, and the peak at 3,329.24 cm^–1^ belongs to the amino group, and peaks at 1,082.15 and 1,158.47 are related to the stretching vibrations of the carboxyl group of chitosan. FTIR also showed characteristic peaks of thymol. The peak at 2,919.19 cm^–1^ belongs to the vibration of C-H in the benzene ring, the absorption peaks at 1,630.90, 1,460.77, and 1,278.23 cm^–1^ belong to unequal stretching vibrations of the phenolic ring, and the peaks at 1,247.83 and 1,098.26 cm^–1^ are related to the O-H and C-O vibrations of thymol (Hosseini et al., 2013; Davidov-Pardo et al., 2015). The presence of these peaks in TCNPs (Figure 1D) without the formation of covalent bonding indicates the formation of TCNPs. A peak shift and flattening were also observed at 3,370.00 cm^–1^, which are attributed to O-H and N-H group stretching and may have resulted from the interactions between the molecules. These results are similar to the study of Ghaderi Ghahfarokhi et al. (2016), where the synthesis of thyme oil-loaded chitosan nanoparticles showed a peak shift and flattening associated with O-H and N-H group stretching at 3,400 cm^–1^. The morphology of nanoparticles analyzed by SEM and transmission electron microscopy (Figures 1A,C) showed that the nanoparticles were uniform in shape with size ranging from 54 to 250 nm in diameter, which is in correlation with the size of thymol-loaded water-soluble chitosan nanoparticles in the previously reported study (Hu et al., 2009). The average size values of the nanoparticles agreed quite well with those obtained by DLS measurement.

### *In vitro* Antibacterial Activity of Thymol-Loaded Chitosan Nanoparticles

**FIGURE 2:**
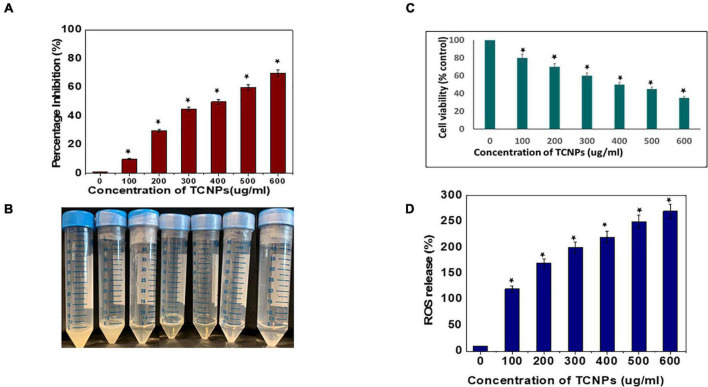
*In vitro* antibacterial activity of the thymol loaded chitosan nanoparticles. **(A)** *ln vitro* antibacterial activity in liquid broth. **(B)** Image of tubes depicting the inhibition visually. **(C)** Percentage of cell viability of the bacterial cells by MTT assay. **(D)** Percentage of reactive oxygen species released. Data are represented as mean ± SD of three independent experiments, each performed in triplicates and considered statistically significant when *p < 0.05.

Antibacterial activity of the synthesized TCNPs was evaluated against the bacterial plant pathogen *Xcc*. The growth of this gram-negative bacterium was inhibited significantly after the application of TCNPs when compared with the untreated control. Furthermore, TCNPs significantly suppressed the growth of *Xcc* in liquid broth cultures within the range from 100 to 600 μg/ml, as shown in Figures 2A,B. Studies have reported that chitosan and thymol exhibit strong antibacterial activity against human and foodborne pathogens (Xu et al., 2008; Orgaz et al., 2011). It is proposed that the charged groups in the polymer backbone of chitosan and the phenolic moieties in the thymol possibly interact with the negatively charged bacterial membrane enhancing the killing efficiency of the phytopathogens (Zivanovic et al., 2005).

### Evaluation of Cell Viability

The potential cytotoxicity of the synthesized TCNPs on *Xcc* cells was assessed by the MTT colorimetric assay. *Xcc* cells were incubated with different concentrations of TCNPs ranging from 100 to 600 μg/ml. The TCNPs were able to reduce the viability of *Xcc* cells in a dose-dependent manner, as shown in Figure 2C. The TCNP concentration of 300 μg/ml was able to reduce the cell viability to 50% and, at higher concentration, exhibited increased cytotoxicity. The cytotoxicity of TCNPs can be attributed to the interaction of the charged groups from chitosan and thymol with the thiol groups of cell wall-bound enzyme resulting in the disruption of the bacterial cell wall, causing cytotoxicity. Similar MTT studies have been reported for both chitosan and thymol individually, where they exhibited cytotoxicity against various bacterial species (Aksoy and Beck, 2017; Khan et al., 2017). It is known that low molecular weight chitosan, apart from exhibiting extracellular antimicrobial activity, can enter cells and disrupt RNA, protein synthesis, and mitochondrial function (Kutawa et al., 2021). Similarly, the charged groups from thymol enter the cell membrane modifying the membrane composition, which causes rupturing of mitochondria leading to cell death (Singh et al., 2017).

### Intracellular Reactive Oxygen Species Generation

It is well known that high levels of Reactive oxygen species (ROS) damages cellular organelles leading to cell death (Liu et al., 2018). Induction of oxidative damage in bacterial pathogens, when exposed to nanoparticles, has been reported in several studies (Tanna et al., 2015; Ogunyemi et al., 2020). To investigate whether TCNPs can enhance the production of ROS in *Xcc* cells, DCFH-DA fluorometric assay was performed in which the magnitude of the fluorescence intensity is proportional to the amount of intracellular ROS generated. The fluorescent intensity of *Xcc* cells significantly increased after treatment with TCNPs in a dose-dependent manner as compared with control (Figure 2D). This indicates that TCNP treatment significantly increased ROS generation in *Xcc* cells. This increased ROS production possibly contributes to the *Xcc* cell death by suppressing adenosine triphosphate generation and DNA replication. The charged groups from chitosan and thymol have been proposed to be the fundamental factor for the interaction with the microbial cell surface resulting in the impairment of bacterial activities (Ramalingam et al., 2016). Generation of ROS and death of bacterial cells have been reported upon treatment with chitosan and thymol during the elimination of preformed biofilms in *Listeria monocytogenes* and *Staphylococcus aureus* (Wang et al., 2019; Ahmed et al., 2020).

### Scanning Electron Microscopy Analysis and Live/Dead Cell Staining

**FIGURE 3:**
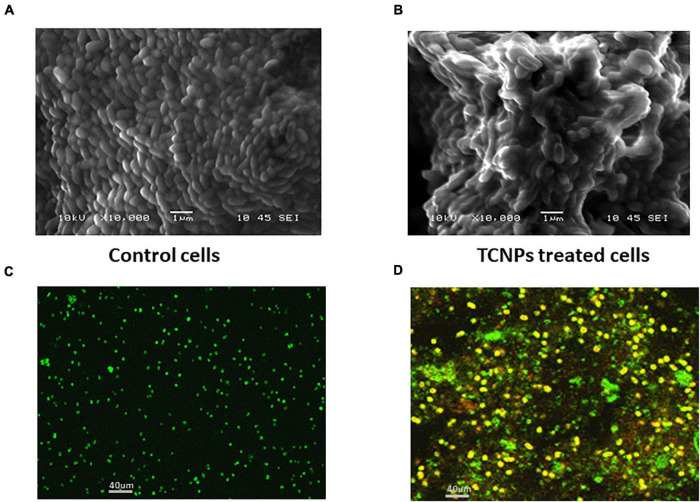
Morphological observation of antibacterial activity of TCNPs. **(A,B)** SEM micrograph for control untreated bacterial cells and bacterial cells treated with TCNPs. **(C,D)** Live dead cell staining by confocal microscopy with green fluorescence denoting live bacteria and yellow/red fluorescence denoting dead bacteria.

To visually characterize the surface morphology and ultrastructural changes of bacterial cells upon exposure to TCNPs, SEM was performed. It was observed that untreated control cells were compact and intact without any changes in the morphology, but after treatment with TCNPs, the bacterial cells were severely deformed with shrinkage in their structure. The cells were fragmented, and the cell membrane ruptured with leakage of the cytoplasmic material, indicating a degeneration in the morphology (Figures 3A,B). Previous studies of SEM analysis of bacterial cells after treatment with chitosan nanoparticles and thymol individually have also shown similar morphological deformation and cell membrane damage against *Xanthomonas* and *Cryptococcus* species (Li et al., 2008; Kumari et al., 2017). Therefore, the underlying mechanism can be attributed to the binding of TCNPs to the bacterial cell surface, increasing membrane permeability, causing leakage of intracellular substances.

To further study the cytotoxicity of TCNPs, a live and dead cell staining assay was performed using a mix of two fluorescent nuclei acid dyes, namely green, fluorescent SYTO9 dye, and red-fluorescent PI dye. PI dye penetrates cells with disrupted membrane and is usually excluded by live cells, whereas SYTO9 dye can enter both live and dead cells. When present together, PI shows a stronger affinity for nucleic acids than SYTO9 and displaces it. A yellow fluorescence can be obtained from dead cells when the replacement of SYTO9 is not completely achieved by PI, and both dyes are present in the cell simultaneously (Stiefel et al., 2015). Confocal imaging of untreated *Xcc* cells exhibited green fluorescence, whereas TCNP-treated cells showed largely red/yellow fluorescence indicative of dead cells denoting damage in the cell membrane (Figures 3C,D). Overall, the results of our study revealed the rupture of the cytoplasmic membrane and internal organization supporting the interaction of TCNPs with the bacterial cell membrane that results in growth inhibition and bacterial death.

### Biofilm Disruption and Reduction of Exopolysaccharides and Xanthomonadin Formation

**FIGURE 4:**
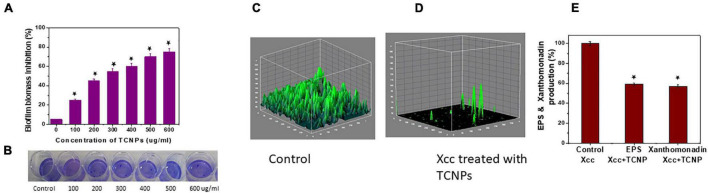
*ln vitro* Biofilm inhibition of Xcc cells. **(A,B)** Biofilm biomass inhibition showing reduction of biofilm growth and the image of crystal violet staining plate. **(C,D)** Confocal images of control untreated biofilm and cells treated with TCNPs denoting reduction in growth. **(E)** Reduction of EPS and xanthomonadin production in control untreated cells and TCNP treated cells. Values are mean ± SD of three independent experiments, each performed in triplicates and considered statistically significant when *p < 0.05.

Biofilm formation in *Xcc* is essential for successful colonization, growth *in planta* and important for its pathogenicity (Rajkumari et al., 2017). Hence, an antibacterial agent should demonstrate inhibitory activity against biofilm growth. A crystal violet assay for biofilm biomass was done to evaluate the ability of TCNPs to disrupt the preformed biofilm. TCNPs displayed significant inhibition on the biofilm formation compared with untreated control in a dose-dependent manner, as shown in Figures 4A,B. Subsequently, the biofilm disruption was further visualized by confocal imaging. Untreated *Xcc* biofilm was uniform and thick (14.2 μm), whereas the TCNP-treated biofilms showed reduced thickness (5 μm) (Figures 4C,D). This demonstrates that the TCNPs are effective in eradicating preformed biofilm and suppressing biofilm formation. The results corroborate with the previous reports of biofilm inhibition *of Pseudomonas aeruginosa* by chitosan nanoparticles and of *Xanthomonas oryzae* by thyme oil (Singh et al., 2017; Khan et al., 2020). Chitosan and thymol are known to suppress the expression of biofilm-associated genes, thus affecting biofilm formation (Singh et al., 2017; Khan et al., 2020).

In most bacteria, EPS production is a crucial factor determining the pathogenesis and viability of the cells. They are considered as an essential component for the virulence and maintenance of cellular integrity (He et al., 2011). In addition, *Xanthomonas* species produce a membrane-bound pigment known as xanthomonadin, which protects it from photodamage and host-induced peroxidation damage (Singh et al., 2017). To better understand the inhibitory action of TCNPs on biofilm, we evaluated the effect of TCNPs on the production of EPS and xanthomonadin. Notably, a significant reduction of EPS and xanthomonadin was observed in the treated cells than the untreated control cells (Figure 4E). This indicates a strong inhibition of EPS and xanthomonadin production in *Xcc* cells by TCNPs, which might directly affect its epiphytic survival and host infection process. The underlying mechanism can be attributed to the affinity of chitosan and thymol to the polymers and DNA in the biofilm, degrading the biofilm matrix (Wang et al., 2019). Similar observations have been reported by various chemical compounds such as thiazole (Liang et al., 2016) and thyme oil (Singh et al., 2017) in blocking the production of EPS and xanthomonadin by interrupting the growth of biofilms in *Xanthomonas* species.

### Mechanism of Antibacterial Action Through Membrane Permeability

**FIGURE 5:**
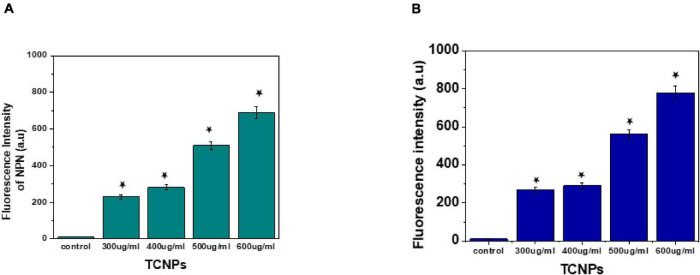
Mode of action by membrane permeability assays. **(A)** NPN assay showing increased fluorescence intensity at sub lethal concentration of TCNPs against Xcc cells. **(B)** Pl assay showing increased fluorescence intensity at sub lethal concentration of TCNPs against Xcc cells denoting membrane damage. Values are mean ± SD of three independent experiments, each performed in triplicate and considered statistically significant when *p < 0.05.

The interaction between nanoparticles and bacteria is best understood by the membrane-targeted mode of action by TCNPs attaching to the bacterial surface. Disruption and permeabilization of *Xcc* cell membrane by TCNPs were evaluated by 1-NPN and PI assays. Outer membrane permeabilization was assessed by the hydrophobic fluorescent probe NPN, which showed increasing fluorescence, indicating the damage in the outer membrane caused by TCNPs (Figure 5A). When the integrity of the outer membrane is disturbed, the lipophilic phase of the cells becomes permeable to the non-polar dye NPN causing an increase in the fluorescence. Furthermore, enhanced PI fluorescence was observed in cells exposed to TCNPs, which indicates that the inner membrane of the *Xcc* cell is compromised when exposed to TCNPs and becomes permeable to PI (Figure 5B). The fluorescence increase of PI was observed in a dose-dependent manner. The results highlighted the fact that with the increasing concentration of TCNPs, there is a corresponding increase in membrane permeabilization of *Xcc* cells. This shows that TCNPs act on *Xcc* by targeting the membrane integrity causing membrane damage. It is proposed that the lipophilic moieties in chitosan and thymol get activated at the lipid bilayer of the cell membrane of bacteria disrupting the cellular membrane and thus increasing the permeability (Liolios et al., 2009). Similar studies with chitosan against *X. axonopodis* pv. *poinsettiicola* also showed induced membrane damage (Chang et al., 2012). The results strongly support that the bactericidal activity of TCNPs involves bacterial membrane disruption.

### Effect of the Thymol-Loaded Chitosan Nanoparticles on the Volatile Signatures of *Xanthomonas campestris* pv. *campestris*

**FIGURE 6:**
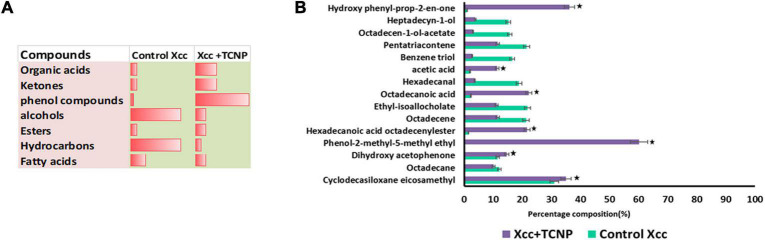
VOC profiling by HS-SPME/GCMS **(A)** Proportion of chemical compounds detected in control untreated Xcc cells and TCNP treated cells. **(B)** Clustered bar plot with changes in the volatile profiles of control untreated Xcc cells and TCNP treated cells. Values are mean ± SD of three independent experiments, each performed in triplicates and considered statistically significant when *p < 0.05.

Microbes emit a plethora of VOCs, which help them to adapt to a particular environment and can act as info chemicals during microbial interactions. Microbial volatiles show a temporal and spatial variation in their responses to environmental conditions (Korpi et al., 2009). We analyzed the VOC profile of *Xcc* cells treated with TCNPs by Headspace/GC-MS. Decreased amounts of carbonyl components and hydrocarbons in the TCNP-treated *Xcc* cells were observed when compared with control cells (Figures 6A,B). In contrast, an increased amount of organic acids and ketones were observed in TCNP-treated *Xcc* cells. Additionally, two phenolic VOCs—phenol methyl methyl ethyl and hydroxy phenyl prop-2-en-one—were observed specifically in *Xcc* cells treated with TCNPs. Similarly, phenolic compounds were seen emitted when *Escherichia coli* was treated with silver nanostructured particles denoting its bactericidal effect (Buszewski et al., 2018; Monedeiro et al., 2021). The various VOC metabolites that are seen perturbed upon TCNP treatment originate from different metabolic pathways. Changes in hydrocarbon, alkanes, and ketones are related to fatty acid and lipid peroxidation pathways, organic acids to glucose metabolism and fermentation, and the phenolic VOCs to phenylalanine metabolism. The cell wall damage and ROS production caused by nanoparticle treatment are known to induce alterations in lipid peroxidation and fatty acid pathways (Monedeiro et al., 2021). The changes in the VOC profile reflect alterations in many metabolic pathways in *Xcc* due to TCNPs. Further studies are needed to identify the novel mechanism involved in the antibacterial activity of TCNPs based on the changes in metabolic pathways observed. Overall, our results demonstrate that the treatment with TCNPs has a direct influence on the VOC profiles of *Xcc* cells.

## Conclusion

In summary, the synthesized TCNPs demonstrated potential bactericidal activity against *Xcc*. The synthesized TCNPs were characterized by standard characterization methods. The characterization confirmed 54–250-nm size of the TCNPs with a uniform shape. The TCNPs exhibited strong bactericidal activity against *Xcc*, which demonstrated a decrease in bacterial growth and cell viability. The evaluated TCNPs also proved as an antibiofilm candidate by inhibiting the biofilm formation and reducing the EPS and xanthomonadin production. Furthermore, ultrastructural studies revealed significant damage to the structure of the bacterial pathogen and wide variation in the cell wall morphology when treated with TCNPs. The cytotoxic activity of TCNPs is mainly due to the disruption of the membrane integrity and reduction of cell viability. Headspace analysis revealed changes in the volatile metabolites produced by *Xcc* when treated with TCNPs. Phenols, organic acids, and ketone volatile compounds were the most abundant compounds released by *Xcc* with TCNPs. This demonstrates that TCNPs have a direct influence on the VOC profiles released as a result of their antibacterial activity. From the present studies, it can be concluded that TCNPs are effective in controlling the *Xcc* growth. The nanoencapsulation can therefore be utilized to produce nanopesticide formulations to control *Xcc* infections in agriculture.

## Data Availability Statement

The original contributions presented in the study are included in the article/Supplementary Material, further inquiries can be directed to the corresponding author/s.

## Author Contributions

SR and SS conceptualized, designed the study, revised the manuscript, and reviewed the final manuscript. SS, NK, and TY carried out the experimental work. SS analyzed, interpreted the data, and drafted the manuscript. All authors contributed to the article and approved the submitted version.

## Conflict of Interest

The authors declare that the research was conducted in the absence of any commercial or financial relationships that could be construed as a potential conflict of interest.

## Publisher’s Note

All claims expressed in this article are solely those of the authors and do not necessarily represent those of their affiliated organizations, or those of the publisher, the editors and the reviewers. Any product that may be evaluated in this article, or claim that may be made by its manufacturer, is not guaranteed or endorsed by the publisher.
