# Supplementary figures and images for: Evaluating the Antibacterial Activity and Mode of Action of Thymol-Loaded Chitosan Nanoparticles Against Plant Bacterial Pathogen Xanthomonas campestris pv. campestris
Source: Front Microbiol. 2022 Jan 14;12:792737. doi: 10.3389/fmicb.2021.792737 (PMC8795685; doi:10.3389/fmicb.2021.792737)

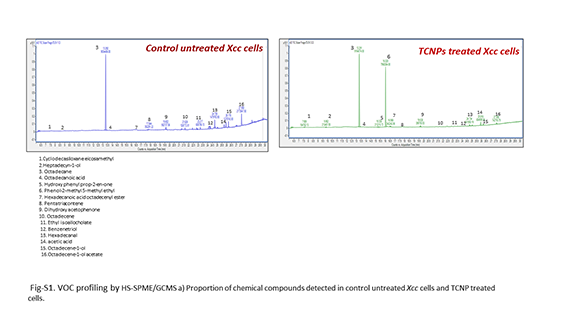

Supplement: Supplementary file 1 [file Image_1.TIF]
